# Software engineers develop better clinical applications than physicists with coding skills

**DOI:** 10.1002/acm2.70687

**Published:** 2026-07-07

**Authors:** Douglas Moseley, Yang Kyun Park, Dongxu Wang

**Affiliations:** ^1^ Department of Radiation Oncology Mayo Clinic Rochester Minnesota USA; ^2^ Department of Radiation Oncology University of Texas Southwestern Medical Center Dallas Texas USA; ^3^ Department of Medical Physics Memorial Sloan Kettering Cancer Center New York New York USA

**Keywords:** AI, medical physics, radiation oncology, vibe coding

## 1 INTRODUCTION

Software applications driven by medical physics have long played an essential role in enhancing efficiency, safety, and quality in radiation therapy. Some of the applications were groundbreaking new platforms, such as treatment planning systems for accurate dose computation in heterogenous media.^1^ Others are scripts that automate clinical workflow or quality assurance.^2^ Many medical physicists write their own scripts or software, while on other occasions, the concepts and algorithms originated from medical physicists are eventually implemented by professional software engineers. Each of these development models carries distinct advantages and limitations. In recent months, the emergence of AI‐assisted coding, or “vibe coding”,^3^ has captivated the imaginations of non‐coders, but also attracted warnings and precautions from the more seasoned developers. In this debate, two colleagues experienced in medical physics clinical application development will explore the merits and challenges of both approaches, with the debate proposition: “*Software engineers develop better clinical applications than physicists with coding skills*.”

Debating for this proposition is Douglas Moseley, PhD, DABR. Dr. Moseley is a faculty physicist at Mayo Clinic in Rochester, Minnesota. Dr. Moseley received his Ph.D. in Applied Mathematics from the University of Western Ontario in 1995. After a 7‐year software development stint at Honeywell Industrial Automation and Control, Doug ended up at the Ontario Cancer Institute within Princess Margaret Cancer Center in Toronto, Canada. He facilitated fundamental research and development of cone‐beam computed tomography with a benchtop kV‐X‐ray system. The translation of this technology to the clinic in 2003 proved the efficacy of image‐guided radiation therapy for photon radiotherapy. Doug's research interests include image‐guidance, tomographic reconstruction and artificial intelligence.

Debating against this proposition is Yang Kyun Park, PhD, DABR. Dr. Park is a board‐certified medical physicist and associate professor of radiation oncology at University of Texas Southwestern Medical Center. He earned his PhD in Radiation Applied Life Science from Seoul National University in South Korea in 2011. Since joining UT Southwestern in 2016, Dr. Park has served in multiple leadership roles within the Division of Medical Physics and Engineering including Associate Director of Medical Physics Residency Program, Associate Director of Computational Physics and Director of Treatment Delivery. Throughout his career, Dr. Park has been dedicated to in‐house software development aimed at automating clinical workflows and improving quality assurance processes. He currently leads development efforts involving the Eclipse Scripting API (ESAPI), overseeing the creation and maintenance of numerous in‐house software tools, either developed directly by him or under his supervision.

## 2 OPENING STATEMENTS

### 2.1 Dr. Moseley, for the proposition

I was a young man of just 22 years when I did a 4‐month co‐op work term in the Accelerator Physics Branch of Atomic Energy of Canada Ltd (AECL) in Chalk River, Ontario. My life changed forever: I met my wife Joanne; I discovered the incredible specialty that is Medical Physics, my future vocation and purpose. And medical linear accelerator designs changed forever as a result of the events that unfolded. Between 1985 and 1987, five radiation therapy patients received massive overdoses when being treated by the Therac25. The Therac25 was a novel multi‐energy medical linear accelerator with a digital control system. Three deaths were directly attributed to these overdoses. This device was designed, manufactured and productized by AECL. I worked with the physicists, engineers, machinists and technicians who designed and built all the bending magnets, waveguides and electron guns that were the key components to this device. In the fall of 1987, this was not a topic of polite conversation at the daily physics coffee.

The error, “Malfunction 54”, is what the machine reported on its digital console. Incredibly, this error was reproduced in testing by East Texas Cancer Center staff members. Physicist Fritz Hager working with the fast fingers of RTT Mary Beth Elliot had uncovered an extremely rare race condition where the entered beam setup parameters confounded the software safety interlock logic in the user interface. Photon mode beam currents were delivered with the target withdrawn. An estimated overdose of 12 500% was delivered in a few electron pencil beam pulses. Imagine if you worked at the clinic where these two patients were killed by this software error. Would you ever trust the equipment again?

The fallout was threefold: 1) The Therac25 was removed from clinical service with the FDA issuing a Class I recall. 2) Beam monitoring and control systems on future linac designs for all manufacturers were made independent from other machine activities such as logging, user interface and communications and 3) The Therac25 incident became a case study in the software engineering literature of “How NOT to develop safety critical software”.

The 1993 review article in IEEE *Computer* 27(7) by Leveson and Turner is a fascinating read. It is 24 pages long and quite thorough in its review of the incident. The authors conclude that attributing these tragedies to a single “software error” or “human error” is dangerously oversimplified and unhelpful. Instead, the accidents were system accidents. They resulted from a complex web of systemic failures across technical, human, and organizational levels. The overarching finding is that relying on software for safety without proper software‐engineering rigor and independent safety mechanisms is fundamentally flawed.

Vibe coders are working as lone coders. This exposes a single point of failure. The Prompt Jockey is responsible for the problem definition and understanding, the application design AND the test cases that are used for unit testing and integration testing. A well‐engineered software system has to test for failures as well as successes. What if unexpected inputs are received? Letters in number fields or vice versa. Robustness and safe failure modes are critical.

When a physicist writes a ‘vibe‐coded’ script, the documentation is usually an afterthought—if it exists at all. The Therac‐25 manual literally defined a fatal, 12 500% overdose with three cryptic words: ‘dose input 2’, and told the operator to press ‘P’ to proceed. Professional software engineers don't just write code; they write comprehensive, hazard‐analyzed documentation. They design error handling that tells the user exactly what failed and prevents them from blindly bypassing a critical safety fault.

Now I don't mean to harsh your vibe. The latest set of LLM's and coding assistants are incredible. They are only going to get better. I believe they should be fully leveraged for retrospective analysis and prototyping. But when it comes time to translate applications to a prospective clinical use where clinical decisions are being made, we need to collaborate with the pros to ensure a transparent, safe and robust system that keeps us and our patients safe.

### 2.2 Dr. Park, against the proposition

Radiation oncology is a multidisciplinary field, and our clinical work depends on a complex mix of people, technology, and processes. Clinical software is now part of that workflow, so it must support safe care, fit the way the clinic actually works, and remain reliable as systems and practices change over time. With that in mind, the key issue in the proposition—“software engineers develop better clinical applications than physicists with coding skills”—is what we mean by “better.” In clinical environments, “better” software is not defined by code elegance; it is defined by safety, clinical accuracy, workflow integration, and long‐term sustainability. Therefore, I cannot agree with the statement.

Even though we have many good commercial products, many institutions still use and develop in‐house software. In‐house software projects are often small‐to medium‐sized, and they are usually made for a specific purpose when we need a solution quickly.^2^ They require strong domain knowledge, because the logic depends on how radiation therapy really works in one's own institution. In many cases, the software also has a relatively short life cycle because workflows change, a TPS/OIS upgrade breaks something, or the clinic adopts a new process. This is a practical reason why in‐house software continues to exist. Importantly, the fact that these tools are local and fast‐moving does not reduce the need for rigor; it shifts the focus to right‐sized controls that fit clinical realities. Even when software engineers write the code, clinical correctness ultimately depends on continuous, embedded physics ownership—otherwise we risk building the wrong thing correctly. In the clinic, physicists with coding skills have an advantage in three dimensions.

First, safety. Software engineers are trained to optimize software correctness and reliability, while physicists carry primary accountability for *clinical semantics* and the consequences of a wrong interpretation. A physicist writing code can identify and prevent semantic errors early in development because they inherently understand the clinical significance of each data element and decision point. Their clinical intuition helps them detect errors that might pass unit tests but fail in real‐world clinical context. In a safety‐critical environment, preventing mistakes during initial development is typically more efficient than detecting them later during review and testing cycles. This idea is consistent with the risk‐based approach in TG‐100, where the goal is to prevent and reduce failure modes in the process, not only to detect errors after they happen.^5^

Second, fitness to local workflow. Physicists understand the local workflow deeply because they live in it every day. TG‐275 shows how important systematic plan and chart review is, and it also reflects how much workflow details matter for safety and efficiency.^6^ Moreover, workflow is not static. It evolves with guidelines, technology, reimbursement, and institutional culture. Developers outside the workflow face a massive “translation cost.” Even if we do our best during the design phase, the design is never perfect from the start. During development, we often discover new edge cases, new workflow needs, or better logic after seeing real data and real clinical cases. If a physicist is coding directly, they can recognize these issues during development and improve the design on the fly. Research on clinical decision support systems has shown that domain expert involvement throughout the data pipeline—not just at the requirements and validation stages—leads to more effective and adopted solutions.^7^

Third, efficient maintenance and updates. In a complex workflow with many outlier cases, it is not realistic to expect perfect performance in every situation, even after commissioning. Clinic workflows continue to evolve, which means in‐house software often requires ongoing updates and re‐validation.^8^ If a tool is developed mainly by a programmer who is not embedded in the clinical workflow, each update cycle faces the “translation cost” repeatedly. In addition, clinical software lives inside regulatory, accreditation, and QA ecosystems, so when ownership is external, adaptation becomes friction. Over time, this friction can discourage frequent updates even when they are needed. When a physicist owns and understands the code directly, updates can be faster, and long‐term maintenance is usually safer and more efficient.

So, who develops better clinical applications? In safety‐critical domains, the optimal model is not outsourcing clinical intelligence to software engineers, but embedding software capability within clinical expertise. I fully acknowledge that large‐scale enterprise systems require professional engineering rigor, and that security, architecture, and scalability benefit from trained engineers. However, most in‐house clinical tools are not global SaaS platforms. They are tightly coupled decision‐support instruments within a specific workflow. In that setting, domain mastery often outweighs engineering abstraction.

## 3 REBUTTALS

### 3.1 Dr. Moseley, for the proposition

Radiation Oncology is an incredible specialty that administers one “drug” to cure cancer: radiation. Where, when and how much is the challenging part. As the first step, we digitize our patients into pixels. These pixels are sorted into piles with segmentation. Pixels are targeted and spared by complex multi‐objective inverse planning systems. Candidate treatment plans are prospectively measured by intricate patient specific dosimetry systems. At the time of treatment, patients are localized with millimeter accuracy by image‐guided positioning systems. And those treatment plans instruct the digital control systems of the RT machines that accelerate particles to nearly the speed of light to deposit therapeutic bursts of energy that work at a molecular level. The digital integrity of these systems and interconnection points are crucial. Without trust, all is lost.

Dr. Park's defense of in‐house development rests on the assumption that clinical safety is fundamentally a problem of semantics. He suggests that a physicist's daily intuition will naturally catch errors that formal engineering unit tests might miss. This is a dangerous paradigm that fundamentally misunderstands how modern software fails. Software in the clinic rarely fails because the underlying radiation physics is misunderstood; it fails structurally. Clinical intuition simply cannot detect memory leaks, asynchronous threading faults, or state machine failures. Arguing that clinical intuition supersedes the need for formal software architecture, defends the exact “cowboy/cowgirl coding” mentality that has historically led to mistreatments.

Furthermore, my opponent frames the “translation cost” between a physicist and a software engineer as an operational friction that hinders progress, championing “on‐the‐fly” development instead. In a safety‐critical medical environment, coding on the fly is not an agile advantage; it is an unregulated liability. That “translation cost” is a vital safety buffer. Forcing a physicist to explicitly translate and document their clinical requirements for an external engineer, prevents the reliance on internalized, undocumented assumptions. A physicist might perfectly code for the specific clinical edge cases they encounter daily but they routinely miss the systemic edge cases. What happens to the application when a network connection drops during DICOM transfer? What is the fail‐safe if a database returns a null value? We do not need physicists to act as amateur developers; we need them to serve as rigorous product owners who define the physical constraints that professional engineers then build upon safely.

Finally, Dr. Park conflates the speed of local maintenance with long‐term reliability. Pushing an update to a local script rapidly because a single physicist “owns” it circumvents the rigorous Verification and Validation (V&V) required for medical devices. When that single author inevitably leaves the clinic, their “efficiently maintained” tool instantly transforms into a black box of technical debt. Nobody else knows how their undocumented logic truly functions. True sustainability is not about how fast one person can push a patch; it is about professional engineering standards like modular design patterns, continuous integration pipelines and regression testing. These goals are achieved by strict version control as well as programming style guides and code review. Fundamental software engineering practices ensure a tool survives its creator.

### 3.2 Dr. Park, against the proposition

In the opening statement, my opponent highlighted an important and painful lesson from the Therac‐25 accidents. I fully agree with the main message that safety‐critical systems require proper software engineering rigor, independent safety mechanisms, and careful attention to failure modes. However, I do not think this example alone is sufficient to conclude that software engineers develop better clinical applications than physicists with coding skills.

Based on the analysis by Leveson and Turner,^4^ several points are relevant. The control system was largely developed by a single programmer or a very small team, with limited formal processes for specification, testing, and independent review. The accidents resulted from a combination of software design flaws and a fundamental misunderstanding of how the clinical workflow would interact with the machine behavior. In addition, overconfidence in software safety led to the removal of hardware interlocks, which might have been avoided with better domain‐informed system design. Error messages were ambiguous and not transparent to end users, which may have been improved with more domain‐informed design. Finally, the rare race condition that triggered the failure would have been difficult to detect with conventional unit testing alone.

From these observations, I think the lesson of this accident is not about who should write the code. Rather, it shows that domain knowledge and clinical understanding are very important in system design, and that independent safety layers are necessary to reduce the impact of software failures. In this sense, the case supports the importance of embedding clinical expertise in the development process.

At the same time, as noted in my opening statement, I am not suggesting that physicists with coding skills should replace professional developers in large‐scale systems such as linac control software or treatment planning systems. For these complex systems, a collaborative model, such as the Michigan model,^9^ where software engineers lead development with strong and continuous physicist involvement, is clearly appropriate.

My opponent also mentioned the risk of a lone “vibe coder” creating a single point of failure. Recently, the concept of “vibe coding”^3^ has been introduced, where users can develop software mainly through prompts and iterative feedback, without deep programming skills. While this approach is still evolving and has not been formally validated in clinical settings, it reflects a general trend of lowering the barrier for domain experts to develop software.

I agree that code developed by a single individual—whether a software engineer or a physicist—without proper review, validation, and documentation can introduce risks in clinical use. However, this risk mainly comes from inadequate processes, not from the coding method itself. AI‐assisted coding does not remove the need for independent review and clinical validation; it only makes the development faster. The same clinical judgment and semantic verification are still required regardless of how the code is generated.

Therefore, for small and well‐defined in‐house applications, even tools developed through prompt‐based approaches can be useful and safe, as long as they go through appropriate independent review, validation, and failure mode analysis, as emphasized in prior work on risk management for clinical software.^2^

In summary, safety and effectiveness in clinical software depend not only on how well the code is written, but also on how well it reflects clinical reality. For many in‐house tools, physicists with coding skills are in a good position to integrate development directly into clinical workflows. Therefore, I cannot conclude that software engineers—even when collaborating with clinicians—necessarily develop better clinical applications.

## AUTHOR CONTRIBUTIONS

DW proposed the debate proposition and edited the article. DM and YKP contributed to writing and editing.

## CONFLICT OF INTEREST STATEMENT

The authors declare no conflicts of interest.
